# Increased expression of the ectoenzyme CD38 in peripheral blood plasmablasts and plasma cells of patients with systemic sclerosis

**DOI:** 10.3389/fimmu.2022.1072462

**Published:** 2022-12-21

**Authors:** S. Agarbati, D. Benfaremo, N. Viola, C. Paolini, S. Svegliati Baroni, A. Funaro, G. Moroncini, F. Malavasi, A. Gabrielli

**Affiliations:** ^1^ Department of Clinical and Molecular Sciences, Marche Polytechnic University, Ancona, Italy; ^2^ Clinica Medica, Department of Internal Medicine, Azienda Ospedaliero Universitaria delle Marche, Ancona, Italy; ^3^ Immunologia Clinica, Department of Internal Medicine, Azienda Ospedaliero Universitaria delle Marche, Ancona, Italy; ^4^ Department of Medical Sciences, University of Turin, Torino, Italy; ^5^ Fondazione Ricerca Molinette, Torino, Italy

**Keywords:** CD38, systemic sclerosis, scleroderma, plasma cells, plasmablasts, B cells, T cells, NK cells

## Abstract

**Objective:**

CD38 is a type II glycoprotein highly expressed on plasmablasts and on short- and long-lived plasma cells, but weakly expressed by lymphoid, myeloid, and non-hematopoietic cells. CD38 is a target for therapies aimed at depleting antibody-producing plasma cells. Systemic sclerosis (SSc) is an immune-mediated disease with a well-documented pathogenic role of B cells. We therefore analyzed CD38 expression in different subsets of peripheral blood mononuclear cells (PBMCs) from a cohort of SSc patients.

**Methods:**

Cell surface expression of CD38 was evaluated on PBMCs from SSc patients using eight-color flow cytometry analysis performed with a FacsCanto II (BD). Healthy individuals were used as controls (HC).

**Results:**

Forty-six SSc patients (mean age 56, range 23-79 years; 38 females and 8 males), and thirty-two age- and sex-matched HC were studied. Twenty-eight patients had the limited cutaneous form and eighteen the diffuse cutaneous form of SSc. The mean disease duration was 7 years. Fourteen patients were on immunosuppressive therapy (14 MMF, 5 RTX). The total percentages of T, B and NK cells were not different between SSc and HC. Compared to HC, SSc patients had higher levels of CD3+CD38+ T cells (p<0.05), higher percentage (p<0.001) of CD3+CD4+CD25+FOXP3+ regulatory T cells, lower percentage (p<0.05) of CD3+CD56+ NK T cells. Moreover, SSc patients had higher levels of CD24^high^CD19+CD38^high^ regulatory B cells than HC (p<0.01), while the amount of CD24+CD19+CD38+CD27+ memory B cells was lower (p<0.001). Finally, the percentages of circulating CD38^high^CD27+ plasmablasts and CD138+CD38^high^ plasma cells were both higher in the SSc group than in HC (p<0.001). We did not observe any correlations between these immunophenotypes and disease subsets or duration, and ongoing immunosuppressive treatment.

**Conclusions:**

The increased expression of CD38 in peripheral blood plasmablasts and plasma cells of SSc patients may suggest this ectoenzyme as a candidate therapeutic target, under the hypothesis that depletion of these cells may beneficially downregulate the chronic immune response in SSc patients. Validation of this data in multicenter cohorts shall be obtained prior to clinical trials with existing anti-CD38 drugs.

## 1 Introduction

Systemic sclerosis (scleroderma; SSc) is an immune-mediated disease of unknown etiology characterized by vasculopathy and fibrosis of the skin and visceral organs, in particular lungs, gastrointestinal tract and heart (1).

Despite all efforts, there is currently no curative therapy and SSc is still a severe disease, causing disability with morbidity and mortality directly linked to the extent of fibrosis (1).

Since immune dysregulation is believed to play a major role in the pathogenesis of SSc, therapeutic regimens based on immunosuppressive drugs like mycophenolate mofetil still remain the main therapeutic option, especially in SSc with severe lung disease (2).

Autoantibodies, some of which potentially pathogenic (3), are a hallmark of SSc and, together with B cell infiltrates in skin biopsies of SSc patients, suggest the involvement of adaptive immunity in the pathogenesis of the disease and fostered the therapeutic use of B-cell depleting drugs such as rituximab (RTX). Rituximab is an anti B-cell antigen CD20 but its mechanism of action in connective tissue diseases is still unclear and probably goes beyond depletion of B cells. Conflicting data have been reported about its efficacy in SSc (4). In a recent systematic review and meta-analysis, Goswami and colleagues showed that RTX, as a treatment of SSc interstitial lung disease, improved FVC and DLco during the first year of treatment (5). Two other systematic reviews and meta-analysis (6, 7) and an observational study which enrolled 254 SSc patients (8) indicated improvement of skin score and stabilization of organ involvement. Despite these observations on possible beneficial effects, long-lasting results have not been documented.

A possible drawback for RTX efficacy may come from the fact that this drug is certainly efficacious in B cell depletion but plasma cells and hematopoietic stem cells are not among its targets. Long-lived plasma cells are thought to be an important player in SSc pathogenesis, as they infiltrate the skin of SSc patients together with CD20+ B cells and being the main source of SSc autoantibodies, that may also have a functional role (9).

CD38, a multifunctional ectoenzyme highly expressed on plasma cells but also on other lymphoid and myeloid cell populations, may be pharmacologically attractive for targeting plasma cells in SSc patients, as demonstrated by the beneficial effect obtained by two anti-CD38 monoclonal antibodies, daratumumab and isatuximab, in patients with multiple myeloma. Furthermore, another possible role of this ectoenzyme in the pathogenesis of SSc has been postulated by Shi and colleagues who showed that CD38 is an important promoter of fibrosis *via* nicotinamide adenine dinucleotide (NAD) depletion (10).

Therefore, in light of the hypothesis that plasma cell depletion through CD38 targeting may be therapeutically rewarding in SSc patients, the present study aimed at evaluating the levels of circulating CD38^high^ plasma cells and the expression of CD38 in other peripheral blood mononuclear cells (PBMCs) in a cohort of patients with SSc.

## 2 Materials and methods

### 2.1 Patients

Forty-six consecutive patients with SSc and thirty-two healthy controls (HC) were enrolled. The inclusion criteria were as follows: 1) age > 18 years; 2) fulfilment of ACR/EULAR 2013 classification criteria (10); 3) capability to provide a written informed consent. There were no exclusion criteria.

HC were enrolled among blood donors and were matched to SSc patient for gender and age.

The following SSc features were recorded: gender, age, duration since the first non-Raynaud’s symptom, type and extent of systemic involvement, subsets (limited or diffuse skin disease (11), autoantibody profile, modified Rodnan Skin Score (mRSS), previous and ongoing immunosuppressive therapy.

The study was approved by the local ethics committee (Comitato Etico Regionale delle Marche, n°2020/159) and was conducted in accordance with the Declaration of Helsinki, 5th edition (2000). Written informed consent was obtained from all patients.

### 2.2 Blood samples - Peripheral blood mononuclear cells

Heparinized peripheral blood was obtained by venipuncture and peripheral blood mononuclear cells (PBMCs) were separated on Ficoll-Hypaque gradient (Amersham) and washed twice with PBS before use.

### 2.3 Flow cytometric analysis

Cell surface expression of CD38 was evaluated on total PBMCs from HCs and SSc patients using eight-color flow cytometry analysis performed by FacsCanto II (Becton-Dickinson, Franklin Lakes, NJ).

Single cells were stained according to standard protocols. Briefly, for the surface-staining cells were incubated for 15 minutes at room temperature with optimal dilution of following conjugated monoclonal antibodies (Becton-Dickinson): CD138*FITC, CD24*PE, CD19*PE-Chlorophyll-Protein, HLA-DR *PE-Chlorophyll-Protein, CD25* PE-cyanine 7, CD38* PE-cyanine 7, CD56* PE-cyanine 7, CD27* allophycocyanin, CD4* allophycocyanin-cyanine 7, CD19* allophycocyanin-cyanine 7, CD20* allophycocyanin-cyanine 7, CD38* V450 and CD3 V500.

Subsequently, for intracellular staining, cells were fixed with Reagent A and then permeabilized with Reagent B (Becton-Dickinson); cells were then stained with the conjugated monoclonal antibody FOXP3*PE-Chlorophyll-Protein (Becton-Dickinson) for 15 minutes at room temperature.

A minimum of 500,000 cells per tube were acquired; frequencies of the different subpopulations and CD38 mean fluorescence intensity (MFI) were subsequently calculated by FacsDiva software (Becton-Dickinson).

**Figure 1:**
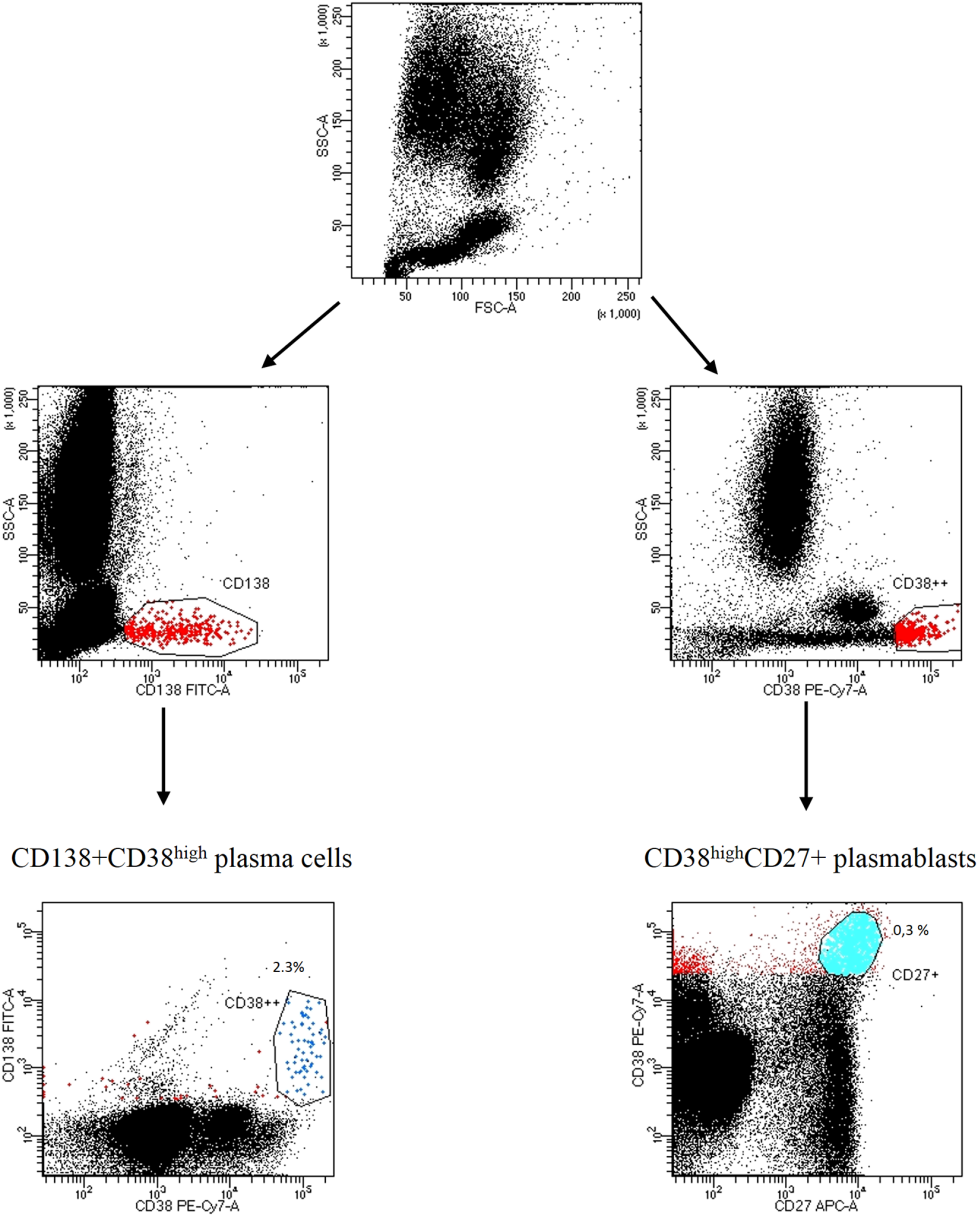
Gating strategy of CD138CD38^high^ plasma cells and CD27+CD38^high^ plasmablasts Representative dot plots from a SSc patient. Lymphocytes were identified and gated according to SSC-A (granularity) vs FSC-A (size). Plasma cells were distinguished by CD138+CD38^high^ expression; plasmablasts were identified by CD27+CD38^high^ expression.

We firstly identified the population of interest gating on CD3-CD56+ NK cells, CD3+CD4+ T helper cells, CD3+CD8+ cytotoxic T cells, CD3+HLA-DR+ or CD3+CD38+ activated T cells, CD3+CD4+CD25+FOXP3+ regulatory T cells, CD19+CD20+ B cells; the expression of ectoenzyme was evaluated on CD24^high^+CD19+CD38^high^ immature B cells, CD24+CD19+CD38+CD27+ memory B cells, CD27^int^CD38^high^ plasmablasts, CD138+ CD38^high^ plasma cells. The gating strategy is shown in **Figure 1**; **Figure S1A, B**.

### 2.4 Statistical analysis

Data was presented with percentages of positive cells and summarized as median. CD38 MFI levels were presented as mean. The differences between groups were compared using the Mann-Whitney test for independent data. Datasets were analysed by Prism 9.0 (GraphPad Software). Statistical significance was set at a level of p <0.05.

## 3 Results

### 3.1 Patients

Thirty-eight females and 8 males (mean age 56, range 23-79 years) composed the SSc group and 32 age- and sex-matched healthy subjects the control group (mean age 49, range 26-71 years; 26 females and 6 males). Clinical and demographic characteristics of the patients are summarized in **Table 1**. Twenty-eight patients had the limited cutaneous form (lcSSc) and eighteen the diffuse cutaneous form (dcSSc) of SSc. The mean disease duration since the first non-Raynaud’s phenomenon symptom was 7 years. Twenty-six patients (56.2%) were anti-topoisomerase I positive, and skin, esophagus and lung were the most affected organs. At the time of sample collection, 14 patients were under active immunosuppressive treatment with MMF (5 of them also received RTX earlier than 6 months before enrolment), while 24 patients (52.1%) had never received immunosuppressive therapy.

**Table 1:** Demographic characteristics of SSc patients and controls.

	SSc (n=46)	Controls (n=32)
Age (years)	56 ± 13.7	49 ± 10.1
Sex (F)	38 (82.6%)	26 (81.2%)
Disease subgroups	lcSSc 28 (61%) dcSSc 18 (39%)	N/A
Mean duration of disease (years)*	7 ± 6.4	N/A
Autoantibodies	ANA† 8 (17.4%) Anti-centromere 9 (19.5%) Anti-topo I§ 26 (56.2%) Other 3 (6.9%)	N/A
Organ involvements	Lung 33 (71.7%) Skin 38 (82.6%) Esophageal 35 (76%) Other 7 (15.2%)	N/A
Mean mRSS	8 ± 10	N/A
Immune-suppressive therapy	*Previous* RTX 4 (8.6%) CYC 4 (8.6%) MMF 4 (8.6%) *Ongoing* RTX° 5 (10.8%) MMF 14 (30.4%)	N/A

Data is presented as mean ± SD or number (%) as appropriate, N/A; not applicable, lcSSc; limited cutaneous systemic sclerosis, dcSSc; diffuse cutaneous systemic sclerosis, †ANA; anti-nuclear antibodies, §anti topo I; anti-topoisomerase I antibodies, mRSS; modified Rodnan Skin Score, RTX; rituximab, CYC; cyclophosphamide, MMF; mycophenolate mofetil.

*from the first non-Raynaud’s symptoms.

° Rituximab was considered ongoing if received < 6 months before the study.

### 3.2 B and T cell compartments in patients with SSc compared to HC

Compared to HC, SSc patients showed no substantial differences in the percentage of CD20+ B lymphocytes (9.6% vs 9%), CD3+ lymphocytes (74.6% vs 73.2%), CD3+CD4+ T helper lymphocytes (48.4% vs 45.7%), CD3+CD8+ T cytotoxic lymphocytes (23% vs 23.2%).

**Figure 2:**
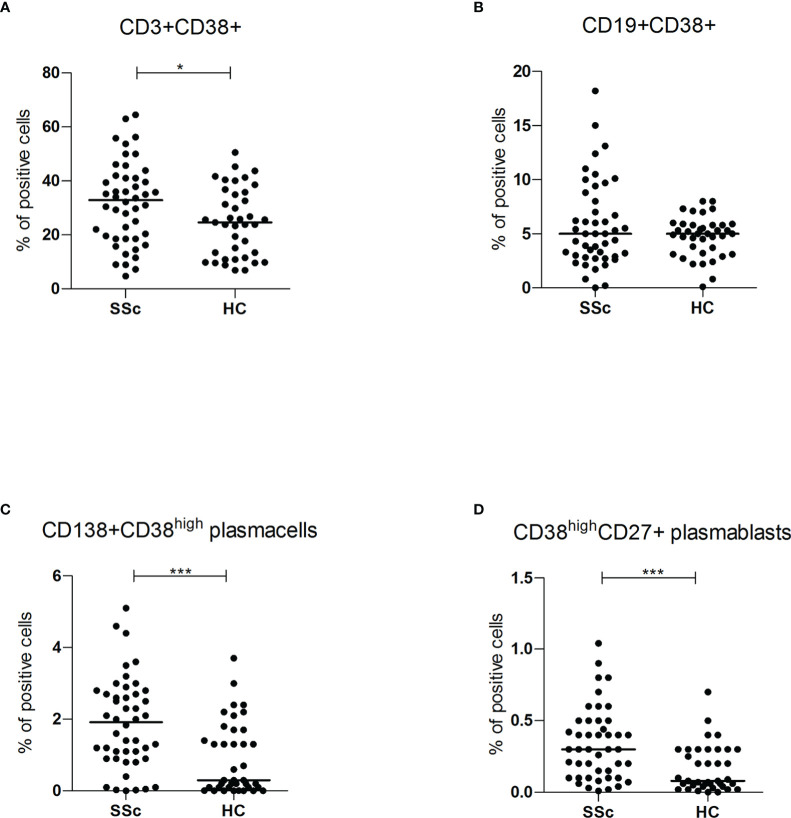
**(A, B)** CD3+CD38+ and CD19+CD38+ surface antigen expression of PBMC from SSc patients (SSc; n = 46) and healthy controls (HC; n = 32) were analyzed by flow cytometry. Results are expressed as percentage of positive cells. Horizontal bars represent median values; *= p < 0.05; *** = p < 0.001. Mann-Whitney test was used for inter-groups analysis; **(C, D)** CD138+CD38^high^ and CD38^high^CD27+ surface antigen expression of PBMC from SSc patients (SSc; n = 46) and healthy controls (HC; n = 32) were analyzed by flow cytometry. Results are expressed as percentage of positive cells. Horizontal bars represent median values; *= p < 0.05; *** = p < 0.001. Mann-Whitney test was used for inter-groups analysis.

However, compared to HC, within the total T lymphocyte population CD38+ cells were more frequent in SSc patients (32.9% vs 24.6%, p<0.05; mean fluorescence intensity (MFI) level 419.1 vs 262.8 p<0.05). No difference was found within the B cell subset (**Figures 2A, B**). No correlation was found between CD3+CD38+ and SSc subsets, disease duration and immunosuppressive treatments (**Figure S2A**).

### 3.3 Plasmablasts and plasma cells in patients with SSc compared to HC

Flow cytometry analysis showed significantly higher levels of both circulating CD138+ CD38^high^ plasma cells (1.9% vs 0.3%, p<0.001) and CD27+ CD38^high^ plasmablasts (0.3% vs 0.1%, p<0.001) in SSc patients compared to HC (**Figures 2C, D**). There was no statistically significant difference in CD38 mean fluorescence intensity (MFI) levels, suggesting an expansion of the CD38^high^ plasma cells and plasmablasts rather than increased expression per cell.

When the levels of CD38+ plasma cells and CD38+ plasmablasts were analyzed in dcSSc and lcSSc, no difference was found in patients with disease duration less or more than 3 years, and in relation to treatment, although HC demonstrated significantly fewer CD38+ cells compared to all subgroups (**Supplementary Figures S2C, D**). Moreover, we observed a non-significant higher trend in the percentage of CD38+ cells in patients with lcSSc compared to dcSSc and in patients not immunosuppressed compared to those immunosuppressed (**Supplementary Figures S2D, C**).

### 3.4 NK and NKT cells in patients with SSc compared to HC

Since it has been suggested that SSc can be triggered by viruses (12) and natural killer (NK) and NKT cells are important effector cells of the innate immune system during infections (13) we investigated the frequency of these cell populations in peripheral blood of patients and controls.

**Figure 3:**
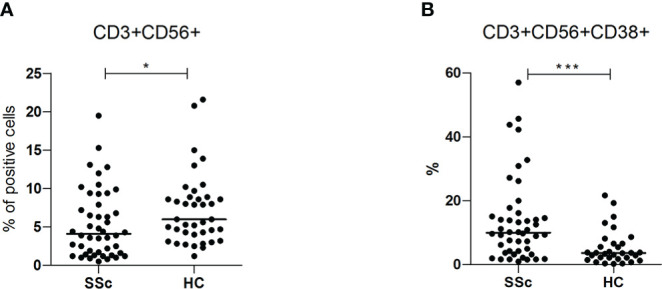
**(A, B)** CD3+CD56+ surface antigen expression of PBMC from SSc patients (SSc; n = 46) and healthy controls (HC; n = 32) and CD3+CD56+CD38+ surface antigen expression of PBMC from SSc patients (SSc; n = 17) and healthy controls (HC; n = 20) were analyzed by flow cytometry. Results are expressed as percentage of positive cells. Horizontal bars represent median values; *= p < 0.05; *** = p < 0.001. Mann-Whitney test was used for inter-groups analysis.

Interestingly, SSc patients showed similar levels of CD3-CD56+ NK cells (11.6% vs 11%) compared to HC, but they had an overall reduced frequency of CD3+CD56+ NKT cells (4.1% vs 6%, p<0.05) with a higher percentage of CD38+ cells within this population (10% vs 3.7%, p<0.001; MFI 434.4 vs 158.6 p<0.0001) (**Figures 3A, B**).

### 3.5 Regulatory T and B cells in patients with SSc compared to HC

**Figure 4:**
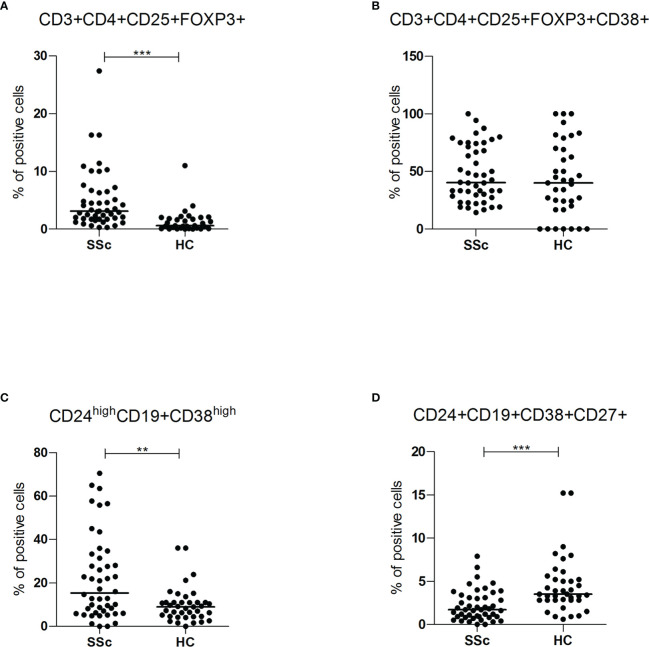
**(A, B)** CD3+CD4+CD25+FOXP3+ and CD3+CD4+CD25+FOXP3+CD38+ intracellular (FOXP3) and surface antigen expression of PBMC from SSc patients (SSc; n = 46) and healthy controls (HC; n = 32) were analyzed by flow cytometry. Results are expressed as percentage of positive cells. Horizontal bars represent median values; ** = p < 0.01, *** = p < 0.001. Mann-Whitney test was used for inter-groups analysis. **(C, D)** CD24^high^CD19+CD38^high^ and CD24+CD19+CD38+CD27+ surface antigen expression of PBMC from SSc patients (SSc; n = 46) and healthy controls (HC; n = 32) were analyzed by flow cytometry. Results are expressed as percentage of positive cells. Horizontal bars represent median values; ** = p < 0.01, *** = p < 0.001. Mann-Whitney test was used for inter-groups analysis.

SSc patients showed increased frequency of CD3+CD4+CD25+Foxp3+ regulatory T lymphocytes (3.1% vs 0.6%, p<0.001), but, in this population, there was no difference in the percentage of cells expressing CD38 (40.2% vs 40%) (**Figures 4A, B**). Regulatory T cells were higher in lcSSc and dcSSc compared to HC, independently of treatment and disease duration (**Supplementary Figure S4A**).

Finally, the subpopulations of regulatory B cells were also investigated. As shown in **Figure 4C, D**, compared to HC, SSc patients showed a higher frequency of CD24^high^CD19+CD38^high^ immature B cells (15.3% vs 9%, p<0.01; MFI 4119 vs 1344, p<0.05) and a lower occurrence of CD24+ CD19+CD38+CD27+ memory B lymphocytes (1.7% vs 3.5%, p <0.001) (**Supplementary Figures S4C, D**). There was no statistically significant difference in CD38 mean fluorescence intensity (MFI) levels of memory B lymphocytes, suggesting a reduction of the CD38 population rather than decreased expression per cell.

Moreover, immature B cells were higher in lcSSc and dcSSc compared to HC, independently of treatment and disease duration. Interestingly, memory B cells were lower than HC in all the subsets analyzed except for SSc patients without immunosuppressive therapy, where the number of these cells were higher than HC.

## 4 Discussion

CD38 is a ubiquitous protein mainly localized on the cell surface and highly expressed in hematopoietic tissues, with an important functional role showing both ADP-ribosyl cyclase and cADPR hydrolase enzymatic activities (14). The significant expression of CD38 in immune cells suggests also a relevant contribution to immune cell homeostasis, as already observed in several inflammatory, neoplastic and immune-mediated diseases (15).

In this study, we have characterized the expression pattern of CD38 on circulating PBMCs of patients with SSc, demonstrating a higher number of CD38+ cells within total T lymphocytes, higher levels of circulating CD38+ plasma cells and plasmablasts, an increased frequency of CD38+ cells within immature B cells and NKT cells in SSc patients compared to HC. Although no difference in the expression of CD38 within total B lymphocytes was observed between SSc and HC, the finding of lower levels in the subgroups treated with immunosuppressive drugs may indicate some effect of these therapeutics on humoral adaptive immunity (16).

Thus, we found that CD38 expression is increased in many peripheral blood cell populations of SSc patients, although its functional role has not been completely defined. Albeit CD38 is usually considered a marker of T cell activation (15), previous studies indicate that its role may be rather multifaceted, ranging from differentiation to effector function and senescence (17).

The data reported herein expand the results by Gumkowska-Sroka et al, who recently reported that SSc patients show a higher frequency of regulatory B lymphocytes, lower levels of memory B cells, NK and NKT cells, and an increase of circulating plasmablasts in patients with SSc compared to HC, that may correlate with disease activity (16). The present study strengthens these findings by demonstrating a higher expression and/or number of CD38+ cells within these populations, paving the way to larger studies supporting the use of CD38 as a target for SSc treatment.

The most significant result of this study is the demonstration of increased levels of circulating CD38^high^ plasma cells and plasmablasts in patients with SSc compared to HC. Several lines of evidence indicate an altered homeostasis of the B cell compartment in PBMCs of patients with SSc (18–21), although the precise role of B cells in the pathogenesis of SSc is still controversial (22–24). In general, B cells of SSc patients are characterized by enhanced pro-inflammatory and pro-fibrotic properties, as well as impaired immunosuppressive activity (25). Activated B cells may contribute to the pathogenesis of SSc by promoting the differentiation of Th2 cells, thereby shifting cytokine production towards cytokines such as IL-6, IL-4, and IL-13, which in turn promote antibody production and tissue fibrosis (23). Furthermore, the discovery of pathogenetic autoantibodies strongly implicate the humoral compartment of adaptive immunity in the development of SSc (26).

Therefore, it is conceivable that B cells may play an important role in the pathogenesis of SSc and other immune-mediated diseases, at least at early stages (27). The partial clinical benefit obtained in SSc patients by depleting B cells with anti-CD20 drug RTX might be explained by lack of expression of CD20 in long-lived plasma cells (28). Having reached the highest degree of differentiation, these plasma cells are able to produce antibodies in the protective niche of the bone marrow, relatively spared from RTX depletion. Thus, an effective immunosuppressive treatment would require elimination of these antibody-producing long-lived plasma cells (4).

The high expression of CD38 in antibody-producing cells suggests its therapeutic use in targeted therapies against immune-mediated diseases like SSc (26, 29). Additionally, CD38 has several important enzymatic activities strongly implicated in the homeostasis of the immune system (30). In fact, over 90% of CD38 acts as an ecto-NADase that catabolizes NAD+. Another role of CD38 is the regulation of extracellular adenosine, which requires consumption of NAD+. The synthesis of adenosine from NAD+ is a complementary mechanism similar to the CD39/CD73-mediated catabolism of ATP to adenosine. Adenosine has an important role in immune modulation because it has been implicated in immune suppression (14). Mechanistically, Shi et al. demonstrated that in SSc the inhibition of CD38 may boosts NAD+ levels, which in turn increases sirtuin (SIRT) activation and, at last, induces the downregulation of TGFβ-SMAD pathway, which is strongly implicated in SSc pathogenesis (28, 31, 32). Thus, our study and the above mentioned reports prompt the use of anti-CD38 therapy in SSc; however, it is unclear whether the best approach would be the use of a cytotoxic monoclonal antibody or a reversible enzymatic inhibitor. Of note, in a recent proof-of-concept study, two patients with systemic lupus erythematosus (SLE) refractory to multiple lines of therapy were successfully treated with anti-CD38 daratumumab, with long-term efficacy that was maintained with belimumab co-administration (33). More recently, Yalcin Mutlu et al. successfully treated a patient with SLE and refractory cerebral vasculitis with daratumumab (34).

Given its peculiar expression pattern on PBMCs, the importance of targeting CD38 in SSc may go beyond the B cell compartment of adaptive immunity. For example, in rheumatoid arthritis CD38 is involved in the regulation of the cellular Treg/Th17 ratio through its expression in NK and NKT cells (35). Therefore, CD38 may also be an important regulator of NK, NKT and T cell lineages and their function, and our finding that CD38 is highly expressed in these populations in SSc suggests an additional potential effect of its drug targeting.

Although we found an increase of CD38+ regulatory B and T cells in this SSc cohort, suggesting a potential limitation of the use of anti-CD38 therapy in patients with SSc, it is thought that these populations are functionally impaired in SSc, thus their role is highly controversial (36–40). Tregs function is thought to be impaired in SSc through mechanisms including reduced production of IL-10 and TGF-β, and reduced plasticity of Treg cells which can differentiate into Th2- or Th17-like cells when exposed to a pro-inflammatory environment (40). Thus, it is not predictable that anti-CD38 treatment would lead to an impairment of the regulatory compartment of adaptive immunity.

Finally, another important issue potentially hampering the safety of CD38 inhibition in SSc is the higher risk of infection and sepsis associated with this treatment as shown by studies in multiple myeloma. The mechanisms through which the inhibition of CD38 increases the infectious risk include not only the depletion of natural and adaptive immune cells (including memory B cells) but also the impairment of several regulatory functions of immune system (41). This suggests the opportunity of further investigating the safety of anti-CD38 cytotoxic antibodies versus reversible CD38 inhibitors.

This study has several limitations. First of all, the sample size is limited and patients are highly heterogeneous with respect to disease subgroups, duration and treatment, which can hinder the interpretation of study results. Importantly, to overcome this potential issue we have conducted stratified analyses taking into account disease characteristics and treatment, although the study was not adequately powered to show differences between groups, if any. The second important limitation is that we did not evaluate the functional role of CD38 in the different compartments of the immune cells, that may be important with regard to the enzymatic activity of this molecule. Therefore, we were unable to provide mechanistic insights into the role of CD38 inhibition in the interaction between PBMCs.

Further studies are therefore needed to validate the pathophysiologic mechanisms of CD38 and to investigate the role of targeted therapies against CD38 in SSc and other immune-mediated diseases.

## Data availability statement

The original contributions presented in the study are included in the article/**Supplementary Material**. Further inquiries can be directed to the corresponding author.

## Ethics statement

The studies involving human participants were reviewed and approved by Comitato Etico Regionale delle Marche, n°2020/159. The patients/participants provided their written informed consent to participate in this study.

## Author contributions

SA and DB drafted the manuscript and designed the figures and tables. SA and DB collected samples from the SSc patients and HC. SA, NV and AF performed the experiments. SA, DB, CP, SS, AF, GM, FM and AG revised the manuscript. SA, DB, GM, FM and AG conceived the topic. All authors contributed to the article and approved the submitted version.
