# Supplementary material for: Increased expression of the ectoenzyme CD38 in peripheral blood plasmablasts and plasma cells of patients with systemic sclerosis
Source: Front Immunol. 2022 Dec 21;13:1072462. doi: 10.3389/fimmu.2022.1072462 (PMC9811259; doi:10.3389/fimmu.2022.1072462)
Supplement: Supplementary file 1 [file DataSheet_1.pdf]

Supplementary Material

Supplementary Figures

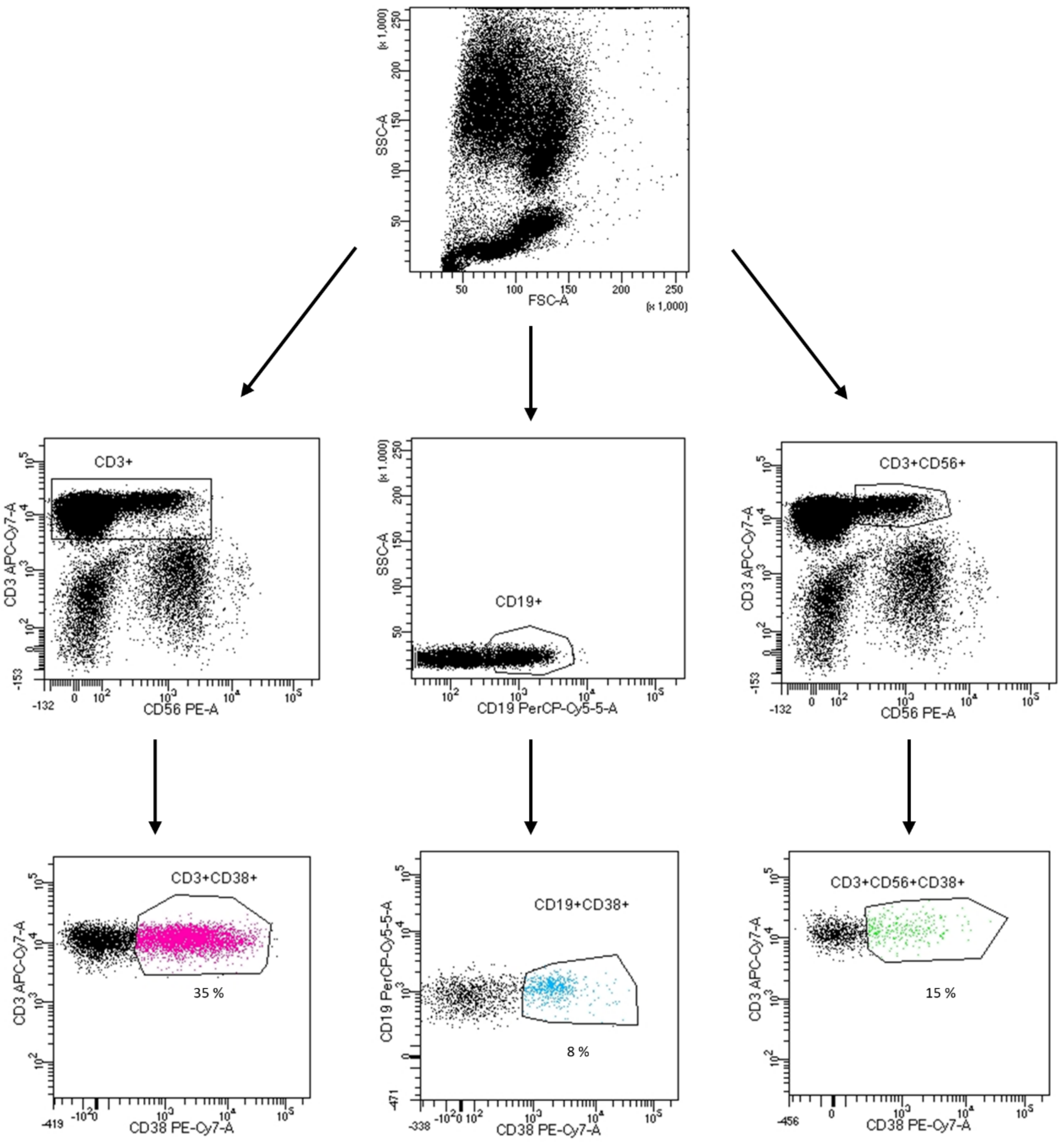

Supplementary Figure 1A. Gating strategy of activated T cells, activated B cells and activated NKT cells.

Representative dot plots from a SSc patient. Lymphocytes were identified and gated according to SSC-A (granularity) vs FSC-A (size). Respectively, activated T cells were distinguished by CD3+CD38+ expression, activated B cells by CD19+CD38+ expression and activated NKT cells by CD3+CD56+CD38+ expression.

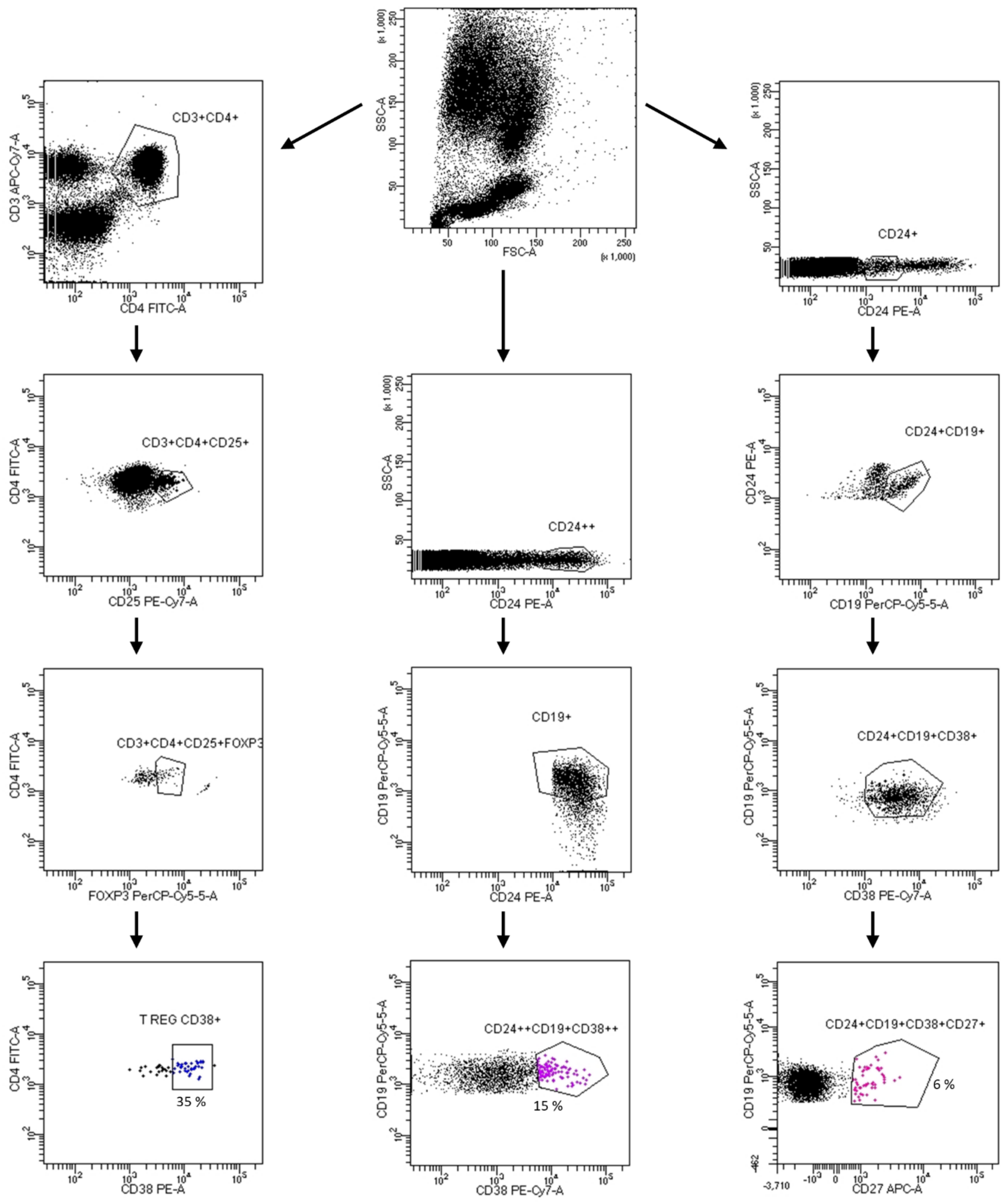

Supplementary Figure 1B. Gating strategy of activated regulatory T cells, immature B cells and memory B lymphocytes.

Representative dot plots from a SSc patient. Lymphocytes were identified and gated according to SSC-A (granularity) vs FSC-A (size). Respectively, activated regulatory T cells were distinguished by CD3+CD4+CD25+FOXP3+CD38+ expression, immature B cells by CD24^{high} CD19+ CD38^{high} expression and memory B lymphocytes by CD24+ CD19+CD38+CD27+ expression.

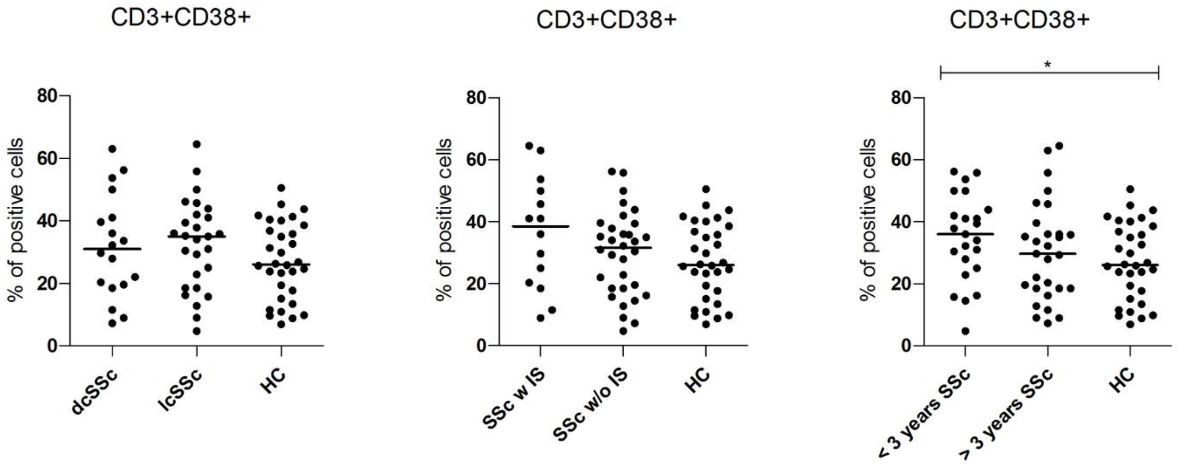

Supplementary Figure 2A. The CD3+CD38+ surface antigen expression of PBMC from SSc patients (SSc; n = 46) and healthy controls (HC; n = 32) analyzed by flow cytometry. SSc patients were stratified for disease subgroups, immunosuppressive therapy at the time of blood draw and disease duration. Results are expressed as percentage of positive cells. Horizontal bars represent median values; *= $p < 0.05$. Mann-Whitney test was used for inter-groups analysis. dcSSC: diffuse SSc, lcSSc: limited SSc; SSc w IS: immunosuppressed SSc, SSc w/o IS: not immunosuppressed SSc; < or > of 3 years SSc: disease duration.

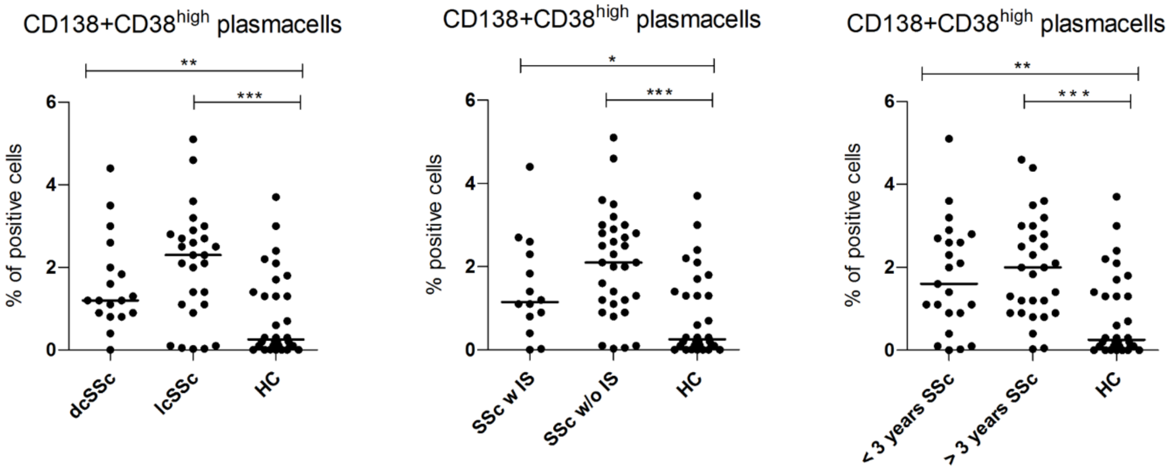

Supplementary Figure 2C. CD138+CD38^{high} plasma cells surface antigen expression of PBMC from SSc patients (SSc; n = 46) and healthy controls (HC; n = 32) analyzed by flow cytometry. SSc patients were stratified for disease subgroups, immunosuppressive therapy at the time of blood draw and disease duration. Results are expressed as percentage of positive cells. Horizontal bars represent median values; *= p < 0.05; ** = p < 0.01, *** = p < 0.001. Mann-Whitney test was used for inter-groups analysis. dcSSC: diffuse SSc, lcSSc: limited SSc; SSc w IS: immunosuppressed SSc, SSc w/o IS: not immunosuppressed SSc; < or > of 3 years SSc: disease duration.

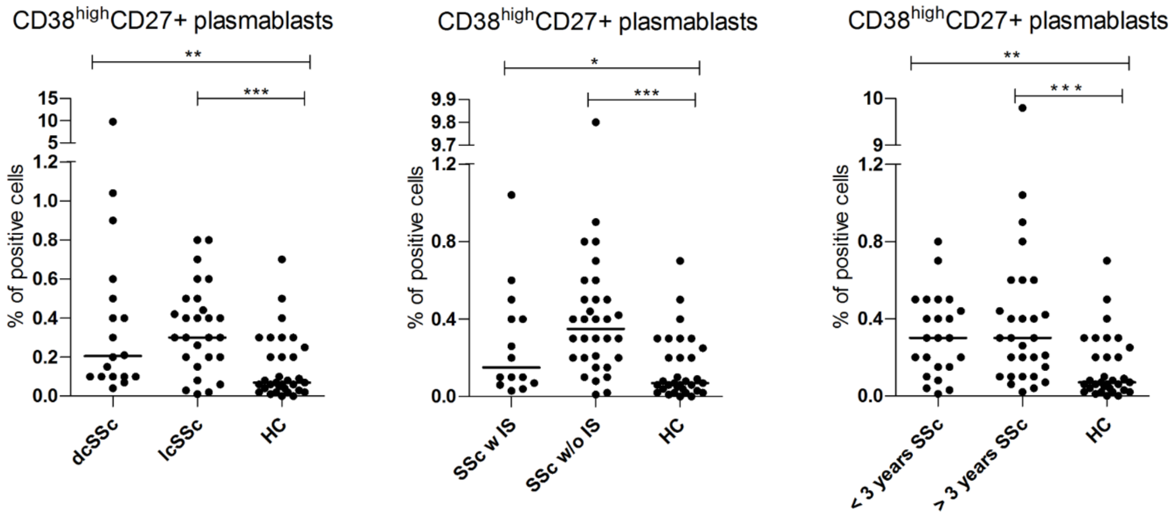

Supplementary Figure 2D. CD138 ^{high} CD27+ surface antigen expression of PBMC from SSc patients (SSc; n = 46) and healthy controls (HC; n = 32) analyzed by flow cytometry. SSc patients were stratified for disease subgroups, immunosuppressive therapy at the time of blood draw and disease duration. Results are expressed as percentage of positive cells. Horizontal bars represent median values; *= $p < 0.05$; ** = $p < 0.01$, *** = $p < 0.001$. Mann-Whitney test was used for inter-groups analysis. dcSSC: diffuse SSc, lcSSc: limited SSc; SSc w IS: immunosuppressed SSc, SSc w/o IS: not immunosuppressed SSc; < or > of 3 years: disease duration.

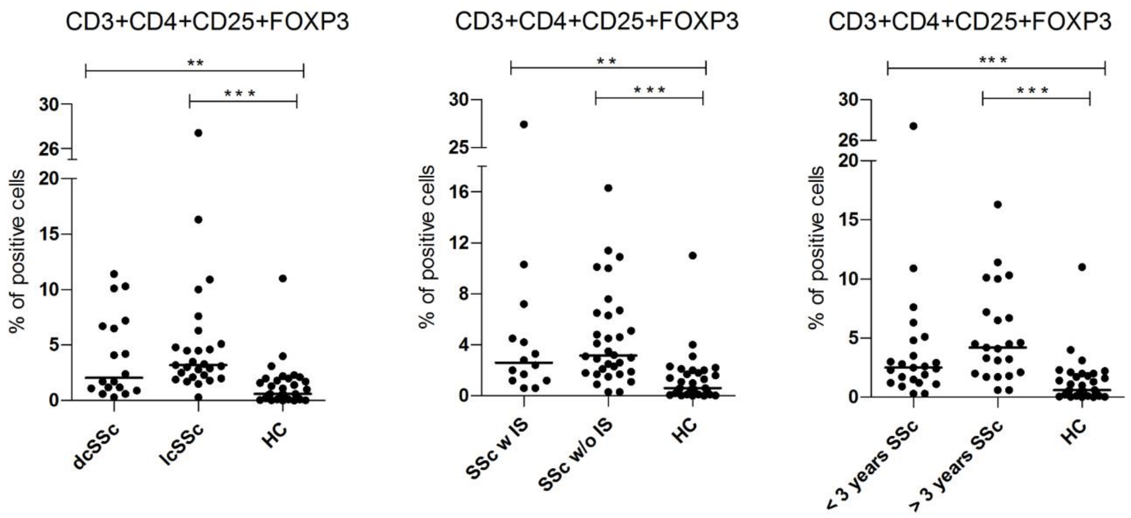

Supplementary Figure 4A. CD3+CD4+CD25+FOXP3+ intracellular (FOXP3) and surface antigen expression of PBMC from SSc patients (SSc; n = 46) and healthy controls (HC; n = 32) analyzed by flow cytometry. SSc patients were stratified for disease subgroups, immunosuppressive therapy at the time of blood draw and disease duration. Results are expressed as percentage of positive cells. Horizontal bars represent median values; ** = p < 0.01, *** = p < 0.001. Mann-Whitney test was used for inter-groups analysis. dcSSC: diffuse SSc, lcSSc: limited SSc; SSc w IS: immunosuppressed SSc, SSc w/o IS: not immunosuppressed SSc; < or > of 3 years SSc: disease duration.

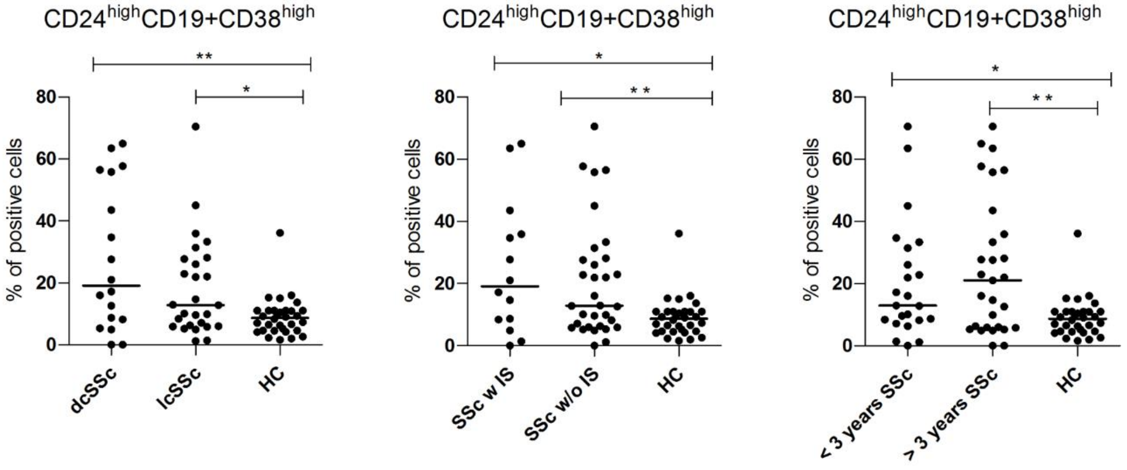

Supplementary Figure 4C. CD24^{high}CD19+CD38^{high} surface antigen expression of PBMC from SSc patients (SSc; n = 46) and healthy controls (HC; n = 32) analyzed by flow cytometry. SSc patients were stratified for disease subgroups, immunosuppressive therapy at the time of blood draw and disease duration. Results are expressed as percentage of positive cells. Horizontal bars represent median values; *= $p < 0.05$; ** = $p < 0.01$. Mann-Whitney test was used for inter-groups analysis. dcSSC: diffuse SSc, lcSSc: limited SSc; SSc w IS: immunosuppressed SSc, SSc w/o IS: not immunosuppressed SSc; < or > of 3 years SSc: disease duration.

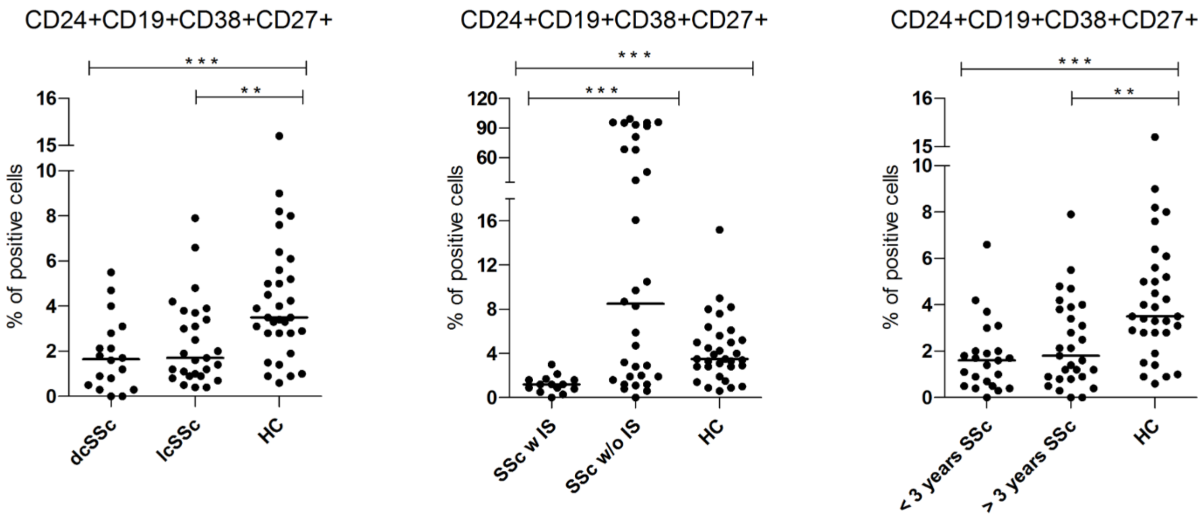

Supplementary Figure 4D. CD24+CD19+CD38+CD27+ surface antigen expression of PBMC from SSc patients (SSc; n = 46) and healthy controls (HC; n = 32) analyzed by flow cytometry. SSc patients were stratified for disease subgroups, immunosuppressive therapy at the time of blood draw and disease duration. Results are expressed as percentage of positive cells. Horizontal bars represent median values; ** = p < 0.01, *** = p < 0.001. Mann-Whitney test was used for inter-groups analysis. dcSSC: diffuse SSc, lcSSc: limited SSc; SSc w IS: immunosuppressed SSc, SSc w/o IS: not immunosuppressed SSc; < or > of 3 years SSc: disease duration.
